# The Effect of Group Discussion on the Quality of Life and HbA1c Levels of Adolescents With Diabetes

**DOI:** 10.5812/ircmj.21110

**Published:** 2014-08-05

**Authors:** Mohamad Afshar, Robabe Memarian, Esa Mohammadi

**Affiliations:** 1 Department of Nursing, Faculty of Medical Sciences, Tarbiat Modares University, Tehran, IR Iran

**Keywords:** Focus Groups, Diabetes Mellitus, Adolescents, Quality of life, Glycosylated Hemoglobin A

## Abstract

**Background:**

Diabetes is a metabolic syndrome and the most common endocrine disorder in childhood and adolescence. Diabetes occurs at any age but the highest outbreak is during ten to 15 years of age and 75% of the cases are diagnosed at the age 18.

**Objectives:**

This study aimed to investigate the effect of a group discussion on the quality of life (QOL) and glycosylated hemoglobin A (HbA1c) levels of adolescents with diabetes.

**Patients and Methods:**

This quasi-experimental study was performed on 56 adolescents with diabetes who were referred to Golabchi Diabetes Center in Kashan, Iran. After obtaining written informed consent from the patients, blood sample was drawn for measuring sugar and HbA1c levels. The participants completed the questionnaire regarding the QOL. Patients were randomly allocated to four groups. All the groups attended similar group discussion sessions, which were conducted according to the guidance of diabetic specialists. The groups’ members followed the discussed instructions for four months. Then, another questionnaire was completed and blood sugar and HbA1c levels were measured again. The results were compared by paired-samples t-test and Wilcoxon signed-rank test.

**Results:**

After the group discussion sessions, in 56% of the patients the HbA1c levels (8.45 ± 1.35 and 6.98 ± 0.89 before and after intervention, respectively) and QOL were improved significantly. The mean age of these patients was 14.75 ± 1.80 years and the mean of daily insulin injection was 35.70 ± 13.42 units.

**Conclusions:**

Sharing experiences trough group discussions and receiving instructive feedbacks can improve the QOL and metabolic status of adolescents with diabetes.

## 1. Background

Diabetes mellitus (DM) is a metabolic disorder that causes improper metabolism of carbohydrates, lipids, and proteins (1). DM is the most common endocrine disorder of childhood and adolescence, which is prevalent at any ages but the highest prevalence rate is during ten to 15 years of age and 75% of cases are diagnosed by the age of 18 (2). The most common form among children and adolescence is insulin-dependent or type 1 DM that encompasses 5% to 10% of all diagnosed cases. The prevalence rate widely varies worldwide and it is rapidly increasing (2). The World Health Organization (WHO) has indicated that in 2000, there were 171 million patients with DM out of which 2103000 cases were from Iran. Moreover, there will be 350 million cases by 2030 out of which 6421000 ones would be in Iran. In addition, based on the recent studies in Iran, the total prevalence rate has been estimated at 6% to 8% out of which 2% belongs to children and adolescents (3).

Adolescence is an influencing period in life and has a significant effect on a person’s life. The management of DM is a great challenge especially in adolescents (4, 5). The adolescents with from type 1 DM are on the verge of being independent from their families but the disease hinders their relations with others (6). Nowadays one of the most significant health problems in developed as well as developing societies is increasing prevalence of DM, which puts financial burden on the affected families and the societies and causes psychologic problems for the patients and families. Studies have shown that the disease can overshadow all aspects of the life of adolescents with DM and can decrease their quality of life (QOL) (7, 8). Therefore, finding some ways to control DM and optimize the QOL is a matter of concern. Based on American Diabetes Association, children > 10 years of age can take the responsibility of the life including how to manage a disease such as DM (9). One of the main ways to increase the QOL and to manage DM is providing proper instructions. The authorities have recognized the importance of self-care approach in patients with DM since 1920, when insulin was discovered (10). Studies have shown that nurses have the key role in providing care for the patients in order to manage the metabolic condition of the patients such as optimizing glycosylated hemoglobin A (HbA1c) levels and to improve the QOL. Nurses shoulder the burden of the main part of the educational program (11) including holding personal or group education and group consultation, establishing educational camps to provide group discussion, furnishing the necessary information via internet and telephone, and holding follow-up sessions such as home visit to give some relevant instructions (12, 13). A study conducted by Delamater et al. showed while psychologic issues in patients with poor control of DM were not considered, advanced DM management strategies including educational programs could not be effective (14). It seems that group discussion will be an appropriate solution to overcome the psychologic problems. Group discussion deals with the relevant issues to discover some solutions to cope with the disease by patients’ cooperation (15). Group discussion enables us to obtain the emotions and experiences of five to 15 persons simultaneously. Spencer et al. suggest it as an ideal approach to investigate individual’s emotions and beliefs about the disease (16).

## 2. Objectives

Adolescence is a transitional stage from childhood to adulthood and is associated with changes in physical and psychologic status. Considering the importance of optimizing the QOL and HbA1c levels of adolescents with DM and preventing the associated psychosomatic and socioeconomic complications due to poor metabolic control, this study was conducted to investigate the influence of group discussion on the QOL and HbA1c levels in adolescents with DM.

## 3. Patients and Methods

This pretest-posttest study was part of an action research conducted on adolescents with DM who were referred to the Golabchi outpatient clinic, Kashan, Iran. This part of the study was conducted from April 6, 2013 to October 22, 2013. Those with 12 to 18 years of age who were diagnosed with DM for at least one year and were registered at the mentioned clinic were included.

The Golabchi outpatient clinic is the largest referral DM center in Kashan, which is governed by Kashan University of Medical Sciences. About 2000 people with DM are registered in this center, which provides patients with some irregular services and training programs. Through reviewing the files of all the registered patients, 58 adolescents with DM were selected according to the inclusion criteria and were invited to participate in this study using a census method. Two patients were excluded; one patient passed away in the first month of the study and the other one withdrew from participation. Finally, 56 patients completed the study.

The participants were invited to the study through telephone call. They were asked to participate in the sessions with one of their parents. After obtaining the informed consent, they took part in a briefing session to participate in group discussion. Blood samples were obtained to measure fasting blood sugar (FBS) and HbA1c levels. FBS and HbA1c were measured using a BT3000 apparatus (made in Italy). FBS was measured through enzyme calorimetric (GOD-PAP) method and the cutoff point of 70 to 115 mg/dL and 140 mg/dL (17) were considered as normal and favorable levels for the DM, respectively. For measuring FBS, TruCal U standard glucose was used to calibrate the apparatus. HbA1c was also measured using enzyme calorimetric method and PT-A1C Cal was used to calibrate the apparatus. The cutoff point of 7% (17) was considered to be normal for HbA1c and higher values were considered as abnormal result. All the blood tests were performed in the reference laboratory of Kashan University of Medical Sciences and by a specific technician.

The participants’ QOL was assessed using a developed and validated questionnaire (18). As the questionnaire was developed and validated for adult patients, we reassessed its validity and reliability for adolescents with DM. Fifteen experts in the field of QOL confirmed the validity of the questionnaire. To determine the reliability, ten adolescents with DM completed the questionnaire with two week interval and then using alpha Cronbach test, the reliability was calculate at 0.84. The questionnaire encompassed five questions on demographic data (i.e. sex, duration of DM, the number of insulin shots per day, the doses, and the prescribed form of insulin) and 39 items on the QOL, which were divided into two groups, general QOL (12 items) and specific QOL (27 items). Among the 39 items, 27 were answered on four point Likert scale from “never = 1” through “always = 4” and 12 were answered with reverse scores, i.e. “never = 4” through “always = 1”. Moreover, there were two general questions as follows: (A) "How do you evaluate your QOL?” (choices: excellent, good, moderate, or bad); and (B) “How would your life have been if you were not affected by DM?” (choices: extraordinary, much better, slightly better, and not different). The participants could obtain the scores 12 through 48 for the general part and 27 through 108 for the specific part of the questionnaire. Overall score ranged between 39 through 156. The higher the score was, the higher the QOL would be. Then, the QOL scores were categorized as good (> 130), moderate (130-110), or weak (< 110) (18).

### 3.1. The Intervention

After the briefing session, the 56 adolescents with DM completed the QOL questionnaire and blood samples were obtained for measuring FBS and HbA1c. Then they were randomly allocated to four 14-individual groups in order to participate in the group discussions. A DM specialist held the two-hour group discussion sessions. The participants could share their problems and experiences with each other. A pamphlet containing related subjects was also available. The DM specialist facilitated the sessions and provided some reinforcements, i.e. amendments (if necessary) and instructions, after the participants shared their experiences at the end of sessions. After completing the group discussions, they followed the learned instructions for the next four months while they were under the supervision of the specialist through phone calls or direct visits to the DM Center. At the end of four months, they completed the QOL questionnaire for the second time and FBS and HbA1c levels were measured again.

### 3.2. Ethical Considerations

The Tarbiat Modares University Review Board and Research Ethics approved the study protocol Committee (No. 30/19101, issued on 18 October 2012). All the participants and their parents were informed that participation in the study would be voluntary and were assured that their personal information would be treated confidentially. All patients and their parents signed the written informed consent before participation in the study.

### 3.3. Data Analysis

Data analysis was performed using SPSS v.16.0 (SPSS Inc, Chicago, IL, USA). There was no missing data. Descriptive statistics were used to describe the patients and disease characteristics. Shapiro-Wilk test was employed to test the normal distribution of the data. The distribution of the QOL scores was normal; therefore, paired-samples t test was used to check the statistical difference between the QOL scores before and after the intervention; however, the distribution of FBS and HbA1c levels before and after the intervention was not normal. Therefore, Wilcoxon signed-rank test was used to check the statistical difference between the FBS and HbA1c levels before and after the intervention. A P value < 0.05 was considered as statistically significant.

## 4. Results

In terms of demographic characteristics, the findings demonstrated that the mean age of patients was 14.75 ± 1.80 years and 42 patients were female. The mean duration of being diagnosed with DM was 4.10 ± 1.79 years (median, 4; IQR, 2.25-5). They were secondary or high schools students. They used NPH and regular insulin shots with the mean dose of 35.70 ± 13.42 units (median, 31; IQR: 25.75-41.5). The patients received at least one to three shots a day and 90% reported two shots a day.

Before participating in the session and in response to two general questions regarding QOL, 59% of the patients reported that they had weak or moderate QOL while 41% indicated good or excellent one; however, after conducting the sessions, 91% stated that they had good or excellent QOL and only 9% had weak QOL. Using paired-samples t-test, there was a significant improvement in the QOL after the group discussion sessions (P = 0.001). These findings showed the beneficial effect of the intervention on the target group.

Before participating in the group discussion, 59% of the patients perceived their own QOL as being weak or moderate and 41% perceived it as good or excellent; however, the rate of perceived good or excellent QOL increased to 91% at the end of the study. Before holding the session and concerning the second question about the QOL in the case of not being affected by DM, 24% responded that they had much better or extraordinary life and 76% replied that they had slightly better or not different one; however, after the sessions, 87% responded that they had much better or extraordinary and 13 replied that they had better or not different. Table 1 indicates that the discussion sessions could improve both specific and general parts of QOL in adolescents with DM.

The first blood test showed that the FBS levels were not in normal range and no one had an FBS < 190 mg/dL. The second blood work showed that after attending the group discussion sessions and following the instructions, the participants FBS levels was changed to favorable range. Wilcoxon signed-rank test showed that the difference between the FBS levels before and after the intervention was significant (Table 2). Moreover, Wilcoxon signed-rank test showed that the HbA1c levels of the subjects were improved in the second assay (P < 0.001) (Table 2).

**Table 1.** Comparing the Quality of Life of Adolescents With Diabetes Before and After Group Discussion Sessions ^a^

Variable	Before Group Discussion	After Group Discussion	P Value
**Specific Quality of Life **	70.62 ± 12.30	95.80 ± 8.21	< 0.001
**General Quality of Life**	35.37 ± 5.33	43.57 ± 3.52	< 0.001
**The Overall Quality of Life**	106 ± 15.95	139.35 ± 10.72	< 0.001

^a^ wall data presented as mean ± SD

**Table 2.** The Median and Interquartile Range of FBS and HbA1c Levels in Adolescents with DM Before and After Group Discussion Sessions ^a^

Variable	Before Group Discussion	After Group Discussion	P Value
**Fasting Blood Sugar **	156 (240-120)	116 (176.25-95.25)	0.002
**HbA1C level**	8 (9.2-7.5)	7 (7.5-6.27)	0.001

^a^ wall data presented as median and interquartile range.

## 5. Discussion

Our results showed that the group discussion had improved the adolescents’ QOL. DM is among the diseases that the patients have the responsibility for the treatment and care. Therefore, providing the patients with DM with opportunities for sharing experiences and receiving instructive feedbacks is of great importance to increase the patients’ QOL. Our findings demonstrated that the QOL of the adolescents with DM was improved after group discussions with instructive feedbacks.

Although adolescence is a period of life when coping with the diseases is hard (19), a group discussion with positive instructive feedbacks can provide an opportunity for the patients to positively cope with the condition and would improve the QOL consequently. Mlynarczyk investigated the association of perceived parental support and different parenting styles with adherence to DM management, metabolic control, and perceived QOL in adolescents who were diagnosed with type 1 DM. Adolescents experience better management outcomes when adolescents and parents become interdependent by working together to achieve these outcomes (20). Moreover, the results revealed that the instructive group discussions had a key role in the QOL; therefore, it is necessary that a skilled healthcare team hold regular educational programs for adolescents with DM to improve their QOL. A study conducted by Simsek et al. on children and adolescents with DM to compare the QOL based on the researchers and parents’ standpoints and showed that the patients had some problems concerning the QOL, particularly in psychosomatic dimension (21). A study conducted by Shih and Simon stated that the interventions made by health care providers to manage DM had improved the QOL in physical and psychologic dimensions, while social and environmental aspects of QOL had not changed significantly (22). The current research showed that after attending the group discussion, the intervention had significantly affected the QOL in both general and special parts. A study performed by Kashfi et al. revealed that a successful management of DM and improving QOL in adolescents with DM needs a proper knowledge on self-care as well as disease management and novel and interesting approaches may be more effective for youngsters (23). Therefore, group discussion, as a novel teaching strategy, might be useful in enabling adolescents with DM.

The present study showed that the group discussions with instructive feedbacks could improve the FBS and HbA1c levels after four months of follow-up. These findings indicated that by sharing the experiences and receiving proper instructions, adolescents could control blood glucose levels properly. When FBS levels were corrected, HbA1c levels, which show the status of blood sugar for three consecutive months, would be corrected. A study conducted by Shirazi et al. on the effect of a self-care approach through group discussion on self-perception has shown that the program had positive influence on metabolic status of adolescent girls with DM. Their results revealed that the patients’ HbA1c levels were significantly improved after the intervention (24). Syrjala et al. demonstrated that a nutritional regimen lowered HbA1c levels (25). Additionally, Turner et al. showed that group discussion decreased HbA1c levels (26). These finding are in agreement with our results that demonstrated the positive effect of proper education on improvement of the QOL of the adolescents with DM. Findings of the present study indicated that group discussions with instructive feedbacks enabled the patients to provide a healthy condition in terms of physical and psychologic status. Moreover, it enabled them in better management of their metabolic parameters that might prevent long-term complications. It seems that group discussion allows the adolescents to share their own experiences, relevant problems, and solutions. In addition, it enables the healthcare providers to develop some strategies to improve patients’ QOL by increasing their control on their own metabolic condition.

The present study showed that the group discussions had positive effects on the perceived QOL. Before taking part in the group discussion, more than a half of the participants perceived their own QOL as being weak or moderate; however, the rate of good or excellent perceived QOL increased to more than 90% at the end of the study. This means that patients were pessimistic about the quality of their lives before the intervention; however, their perception was improved at the end of the study.

Patients with DM, especially adolescents who have peculiar lifestyle due to their age, should be provided with necessary information regarding the control of HbA1c levels and ultimately managing DM for improving their QOL. In agreement with similar studies, our results demonstrated that group discussion is an effective method to achieve the treatment goals in adolescents with DM.

It has been shown that sharing positive experiences through group discussion is an effective method for empowering patients to cope with their diseases; however, this method has been rarely used in patients with DM. The present study was one of the few studies that used group discussion in adolescents with DM. We also used an indigenous QOL questionnaire that was culturally appropriate for Iranian patients; however, some limitations might affect the generalization of the results. Firstly, this study was conducted on adolescents with DM and therefore, the results cannot be generalized to the adults. Moreover, the small sample size and lacking a control group might limit the generalizability of the results. Therefore, further studies with larger sample size and with a control group are suggested.
